# Prehospital intravenous fentanyl administered by ambulance personnel: a cluster-randomised comparison of two treatment protocols

**DOI:** 10.1186/s13049-019-0588-4

**Published:** 2019-02-07

**Authors:** Kristian D. Friesgaard, Hans Kirkegaard, Claus-Henrik Rasmussen, Matthias Giebner, Erika F. Christensen, Lone Nikolajsen

**Affiliations:** 1 Research Department, Prehospital Emergency Medical Service, Central Denmark Region, Olof Palmes Allé 34, 8200 Aarhus N, Denmark; 2 Department of Anaesthesiology, Regional Hospital of Horsens, Horsens, Denmark; 3 Falck Danmark A/S, Central Denmark Region, Aarhus, Denmark; 4 Response A/S, Central Denmark Region, Hedensted, Denmark; 5 Department of Clinical Medicine, Prehospital and Emergency Research, Aalborg University, Aalborg, Denmark; 6 Department of Anaesthesiology and Intensive Care, Emergency Clinic Aalborg University Hospital, Aalborg, Denmark; 7 Department of Anaesthesiology and Intensive Care, Aarhus University Hospital, Aarhus, Denmark

**Keywords:** Prehospital, Acute pain, Intravenous fentanyl, Ambulance personnel

## Abstract

**Background:**

Prehospital acute pain is a frequent symptom that is often inadequately managed. The concerns of opioid induced side effects are well-founded. To ensure patient safety, ambulance personnel are therefore provided with treatment protocols with dosing restrictions, however, with the concomitant risk of insufficient pain treatment of the patients. The aim of this study was to investigate the impact of a liberal intravenous fentanyl treatment protocol on efficacy and safety measures.

**Methods:**

A two-armed, cluster-randomised trial was conducted in the Central Denmark Region over a 1-year period. Ambulance stations (stratified according to size) were randomised to follow either a liberal treatment protocol (3 μg/kg) or a standard treatment protocol (2 μg/kg). The primary outcome was the proportion of patients with sufficient pan relief (numeric rating scale (NRS, 0–10) < 3) at hospital arrival. Secondary outcomes included abnormal vital parameters as proxy measures of safety. A multi-level mixed effect logistic regression model was applied.

**Results:**

In total, 5278 patients were included. Ambulance personnel following the liberal protocol administered higher doses of fentanyl [117.7 μg (95% CI 116.7–118.6)] than ambulance personnel following the standard protocol [111.5 μg (95% CI 110.7–112.4), *P* = 0.0001]. The number of patient with sufficient pain relief at hospital arrival was higher in the liberal treatment group than the standard treatment group [44.0% (95% CI 41.8–46.1) vs. 37.4% (95% CI 35.2–39.6), adjusted odds ratio 1.47 (95% CI 1.17–1.84)]. The relative decrease in NRS scores during transport was less evident [adjusted odds ratio 1.18 (95% CI 0.95–1.48)]. The occurrences of abnormal vital parameters were similar in both groups.

**Conclusions:**

Liberalising an intravenous fentanyl treatment protocol applied by ambulance personnel slightly increased the number of patients with sufficient pain relief at hospital arrival without compromising patient safety. Future efforts of training ambulance personnel are needed to further improve protocol adherence and quality of treatment.

**Trial registration:**

ClinicalTrials.gov (NCT02914678). Date of registration: 26th September, 2016.

## Background

Efficient analgesic treatment is fundamental to ensure patient comfort and to facilitate transport from incident site to hospital [1, 2]. Notwithstanding, several studies have shown that acute pain remains insufficiently treated in emergency settings, which may be even more frequent in an austere and uncontrolled prehospital environment [3, 4]. A recent prehospital investigation has reported a a high prevalence of 43% for insufficient pain relief at hospital arrival among trauma patients treated by experienced physicians [5].

Intravenous opioids are the mainstay in the rapid relief of severe acute pain, but side effects, such as sedation and respiratory depression, cannot be overlooked [6–18]. Due to patient safety, non-physician staff are therefore provided with simple one-drug protocols with dosing restrictions, however, with the potential risk of insufficient pain relief. In a recent prehospital study including 2348 adults and adolescents treated with intravenous fentanyl in a maximum dose of 2 μg/kg by Danish ambulance personnel, we showed that a substantial number of patients (58.4%) had moderate to severe pain at hospital arrival. In the same study, the frequency of abnormal vital parameters, as proxy measures of opioid-induced serious adverse effects, was low [19].

The aim of this study was therefore to explore the impact of liberalising a standard treatment protocol from a maximum dose of 2 μg/kg to an allowed maximum dose of 3 μg/kg on the intensity of pain at hospital arrival. We hypothesised that a larger proportion of patients would experience sufficient pain relief with the liberal treatment protocol. In addition, we examined the frequency of abnormal vital parameters.

## Methods

### Study design

This study was a two-armed, open-label, cluster-randomised controlled trial comparing the impact of a standard and a liberal fentanyl treatment protocol on efficacy and safety parameters.

### Setting

The trial was conducted in the Central Denmark Region over a one-year period from 1st October 2016 to 30th September 2017. Sixty-six ambulances from 36 ambulance stations provide the emergency medical service for the 1,300,000 inhabitants of Central Denmark Region, corresponding to 22% of the Danish population. The Region covers both rural and urban areas [20]. The prehospital model is two-tiered with Emergency Medical Technicians (EMTs) or Paramedics (PMs) doing ground ambulance transport, and prehospital physicians/anaesthesiologists being dispatched to potentially life-threatening conditions in a rendezvous setup (e.g. in case of an opioid overdose and need of advanced airway management). Few stations in the Central Denmark Region also have nurse-manned emergency care units that can be dispatched in cases where physicians are unavailable [21].

All patient care recordings were either automatically (peripheral oxygen saturation, blood pressure and pulse) or manually (Glasgow Coma Scale (GCS)), respiratory rate) entered into an electronic touchscreen-based prehospital medical record by EMTs/PMs. Assessment of pain intensity (numeric rating scale (NRS), 0–10) was required according to protocol from before initiation of fentanyl treatment and every 5–10 min until hospital arrival. Furthermore, each registration was logged with the exact date and time combined with the title (EMT/PM/physician/nurse) and unique user identification number of the treating healthcare provider.

### Population

The sample included all patients treated with intravenous fentanyl by EMTs or PMs. We did not consider patients for analysis if: 1) prehospital physicians were dispatched to the scene and/or were providers of treatment, 2) they were treated by personnel other than EMTs/PMs (nurses), 3) they had unregistered personal civil registration numbers and 4) the same patient appeared more than once in the inclusion period, in which only the first case was included. Patients with pain scores registered at both baseline and hospital arrival were considered for the analysis of efficacy, and all patients were considered for the analysis of safety.

### Randomisation

Randomisation was performed at the ambulance station level with allocation to either the standard treatment protocol for intravenous fentanyl (max 2 μg/kg) or the liberal treatment protocol (max 3 μg/kg). The 36 ambulance stations were stratified into 5 levels based on the average number of transports per month (level 1 (*n* = 8): < 70 transports), level 2 (*n* = 12): 70–199 transports, level 3 (*n* = 8): 200–399 transports, level 4 (*n* = 4): 400–899 transports, and level 5 (*n* = 4): ≥ 900 transports). Each type of treatment protocol was then randomly allocated in a 1:1 ratio within each of the 5 levels. Randomisation of ambulance stations was carried out by an impartial statistician at Aarhus University using STATA version 13.1 (StataCorp, TX, USA).

### Blinding

Once randomised, ambulance station leaders and personnel were instructed to follow the assigned treatment protocol throughout the study period and were thus not blinded. Patients treated with intravenous fentanyl were unaware of the treatment protocol applied.

### Intervention

A liberal fentanyl treatment protocol was implemented in the ambulance stations allocated to the intervention arm with a 2-month run-in period prior to study start between 1st August 2016 and 30th September 2016. The liberal treatment protocol allowed EMTs/PMs to administer intravenous fentanyl at a maximum dose of 3 μg/kg, with the first dose limited to 1.5 μg/kg. The new fentanyl treatment protocol was implemented at the enrolled stations following standard operating procedures and thus left to the discretion of the leaders of the enrolled ambulance stations. For ambulance stations not allocated to the new protocol, the maximum dose of fentanyl was 2 μg/kg with the first dose limited to 1 μg/kg. The exact dosing of fentanyl was decided by the EMT/PM on a μg/kg basis considering patient characteristics such as comorbidity and age.

### Outcomes

Primary and secondary outcomes pertained to the patient level. The primary outcome was the proportion of patients with sufficient pain relief at hospital arrival, defined as an NRS equal to or below 3 [22]. As proxy measures of safety, secondary outcomes included any occurrence of abnormal vital parameters defined as follows: GCS < 15, respiratory rate < 10/min, peripheral oxygen saturation < 90% and mean arterial pressure (MAP) < 70 mmHg [calculated as ((2 x diastolic blood pressure) + (systolic blood pressure))/3] [23]. To investigate whether the mean cumulative dose of fentanyl increased following change of protocol, fentanyl administration practices for each EMT/PM was assessed for a one-year period prior to study start between 1st August 2015 and 31st July 2016.

### Data analysis

Information regarding clinical outcomes was extracted from the electronic medical record. Dispatch data on prehospital time stamps and geographical information on each transport were obtained from the technical software used by the dispatch staff of the Emergency Medical Communication Center [21]. Individual data linkage across registries and exact information on age and sex was enabled by each patient’s central personal registration number [24]. The Danish National Patient Registry were used to obtain the patients’ primary hospital diagnoses classified according to the Danish version of the International Classification of Diseases, 10th revision (ICD-10) [25] and to calculate a 10-year Charlson Comorbidity Index (CCI) score [26, 27]. Primary hospital diagnosis codes were considered reasonable for linkage with electronic medical record data if patients were admitted within 12 h of prehospital (EMCC) contact.

All statistical analyses were conducted using STATA version 13.1 (StataCorp, TX, USA). Means with 95% confidence intervals (CI) are included for continuous parametric variables. Continuous nonparametric data are provided as medians with interquartile ranges (IQR). Categorical and binomial data are presented as numbers and proportions with 95% CIs. A chi square test, unpaired or paired students t-test or Mann-Whitney U-test is used for descriptive statistics when appropriate. A multi-level mixed effect logistic regression model was fitted on binary outcomes in order to account for the potential correlation between patients within the same cluster (i.e. patients nested within EMT/PM and EMTs/PMs nested within ambulance station). The highest cluster level was defined as 1) ambulance station, followed by 2) EMT/PM and 3) the patient. For the adjusted estimates of safety outcomes, the model could not be fitted without removing the highest level of clustering. The results are presented as unadjusted and adjusted odds ratios (OR) with 95% CIs and patients treated with the standard protocol as reference.

The covariates considered in the multivariate statistical analyses are based on the existing literature and clinical experience: age (restricted cubic splines) [5, 28–32], sex (binary) [5, 31], CCI score (0, 1, 2, 3+) [33–35], underlying cause of pain (binary) [28, 29, 36, 37] and patient time with EMT/PM (logarithmic function of time) [29, 31, 32, 38]. On the EMT/PM cluster level, a mean cumulative dose of fentanyl was added as a covariate, and the title of ambulance personnel (EMT or PM) was used as a proxy measure of experience. Ambulance station size (categorical) was added on the highest cluster level. Age and prehospital patient time were categorised as above to ensure the best statistical model fit. The underlying cause of pain was defined by primary hospital discharge codes (ICD-10), and patients were stratified according to ICD-10 chapter 19 (injury, poisoning, and certain other consequences of external causes (yes/no)).

To handle potential differences in baseline pain intensity [5, 28–32, 39] between the standard and the liberal treatment group, we assessed the relative (%) change in NRS from baseline to hospital arrival in a supplemental post-hoc analysis. The relative change in pain scores during ambulance transport was assessed with a multilevel mixed effect ordinal logistic regression model [40], using the same cluster levels and covariates as described above.

### Sample size

We used a previous study from the same prehospital setting and with a similar patient population treated with intravenous fentanyl by EMTs/PMs as reference, in which 42% achieved NRS ≤ 3 at hospital arrival [19]. With a fixed number of clusters (36 ambulance stations), an intracluster correlation at 0.05 [41], 90% power and an alpha level at 5%, at least 1980 patients in each arm would be required in order to identify a 10% points increase in patients with sufficient pain relief (NRS ≤ 3) at hospital arrival in the liberal pain treatment protocol compared to the standard pain treatment protocol. The data analyses are based on complete cases and include no imputation for missing data. Two-sided tests with *P* values < 0.05 were considered statistically significant.

### Ethics

The study was approved by the Danish Data Protection Agency (no. 1-16-02-294-16) and the National Board of Health (no. 3-3013-2002/1). The local ethics committee was consulted, and the study was approved with a waiver of patient consent. The trial was registered at ClinicalTrials.gov (NCT02914678).

## Results

**Fig. 1:**
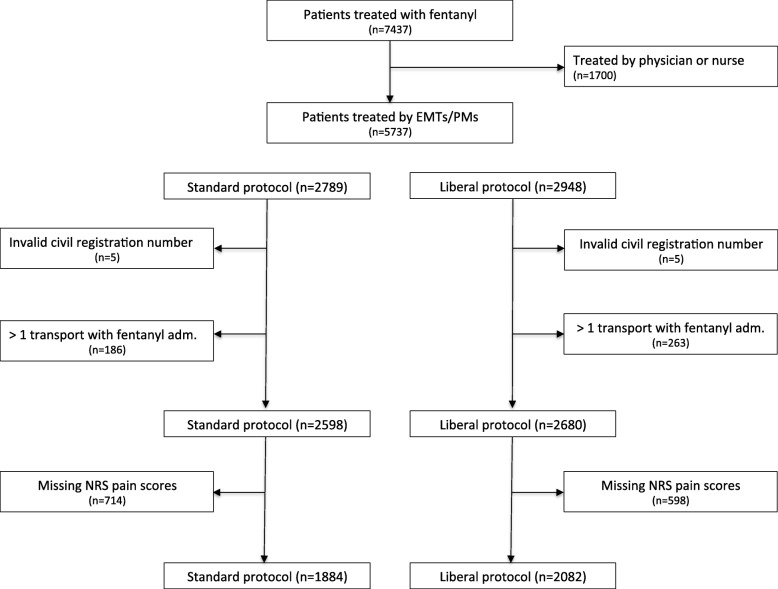
Flowchart for included patients. Abbreviations: EMTs; Emergency Medical Technicians, PMs, Paramedics, NRS; Numeric Rating Scale, adm.; administration

**Fig. 2:**
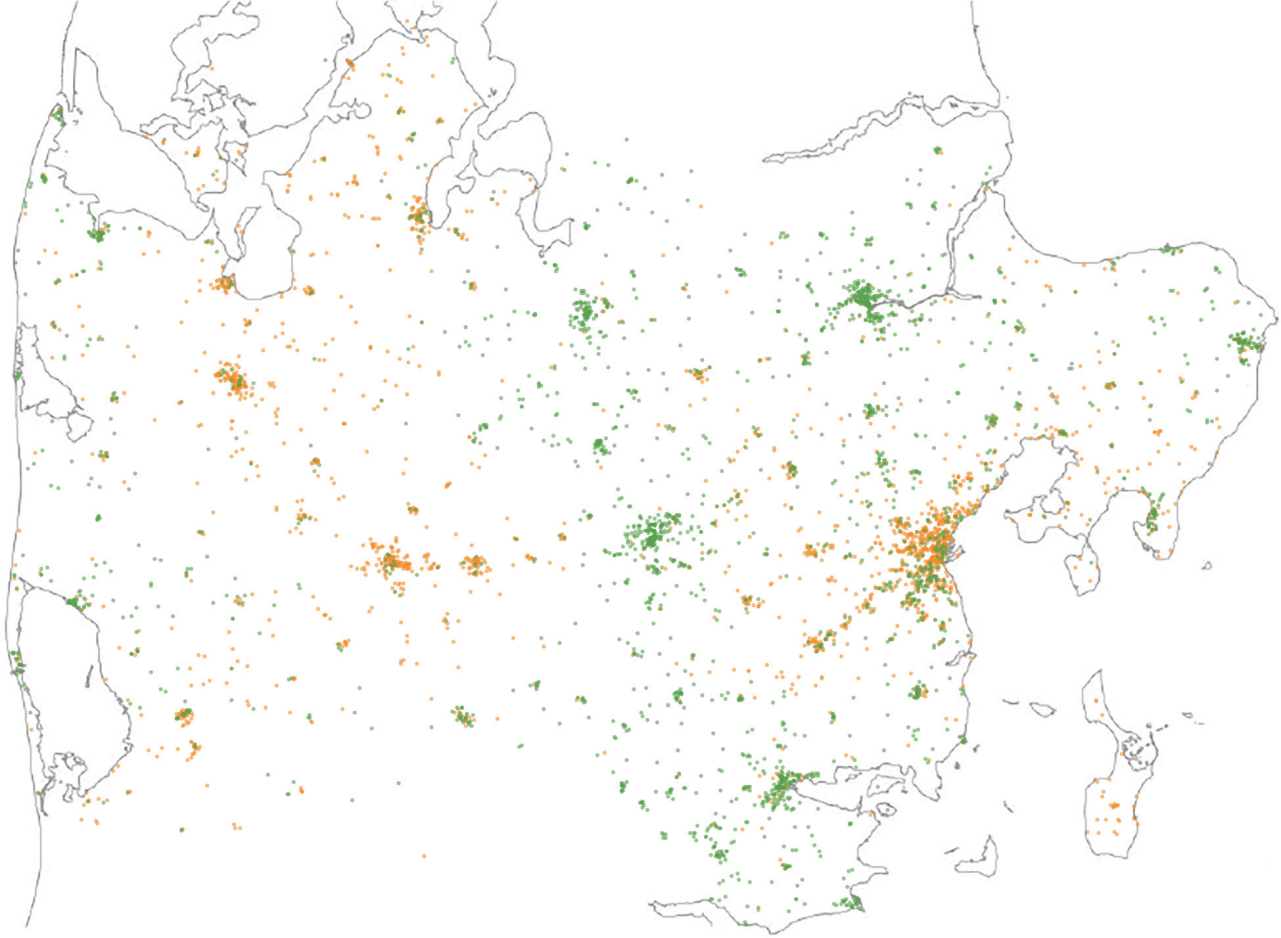
Map of Central Denmark Region with the clusters of included patients. Orange dots = Patients treated according to the standard treatment protocol (max 2 μg/kg). Green dots = Patients treated according to the liberal treatment protocol (max 3 μg/kg)

Patient flow is presented in Figs. 1 and 2 gives an impression of how patients are grouped in clusters. A total of 2598 patients were treated with the standard treatment protocol and 2680 patients were treated with the liberal treatment protocol. No major differences in baseline characteristics of patients were seen (Table 1). As regards the primary outcome, the proportion of patients who had sufficient pain relief at hospital arrival was higher in the liberal treatment group than in the standard treatment group [44.0% (95% CI 41.8–46.1) vs. 37.4% (95% CI 35.2–39.6), *P* = 0.001] (Table 2). Taking covariates and unobserved intracluster correlations into account, the difference remained significant [OR 1.47 (95% CI 1.17–1.84)] (Table 3). In the supplemental post-hoc analysis, there was no difference in the relative decrease in pain scores during transport [43.0% (95% CI 41.3–44.7) vs. 40.5% (95% CI 39.0–42.1)] with an adjusted OR equal to 1.18 (95% CI 0.95–1.48). No apparent differences in the occurrence of abnormal vital parameters were found (Tables 2 and 3) and none of the patients were treated with an antidote (naloxone). A similar number of patients in each group received an anti-emetic (ondansetron) [4.9% (95% CI 4.1–5.8) vs. 4.1% (95% CI 3.4–4.9), *P* = 0.15].

**Table 1:** Baseline characteristics of included patients

Treatment protocol	Standard (2 μg/kg) (*n* = 2598)	Liberal (3 μg/kg) (*n* = 2680)
Age, years (95% CI)	56.3 (55.4–57.2)	56.9 (56.0–57.7)
Male sex, % (95% CI)	45.4 (43.5–47.3)	48.1 (46.2–50.0)
CCI score, % (95% CI)		
0	59.7 (57.7–61.6)	56.9 (55.0–58.8)
1	14.9 (13.5–16.3)	15.0 (13.7–16.4)
2	10.0 (8.9–11.2)	10.9 (9.7–12.1)
3+	15.4 (14.1–16.9)	17.2 (15.7–18.6)
Primary hospital diagnosis code (ICD-10) chapter 19 (injury), % (95% CI):		
Yes	50.3 (48.3–52.2)	47.8 (45.9–49.7)
Cum. dose (μg) fentanyl, mean (95% CI)	111.5 (110.7–112.4)	117.7 (116.7–118.6)
Treated by paramedic, % (95% CI)	9.2 (8.1–10.4)	7.4 (6.4–8.4)
Patient recruitment according tostation size (transports/month), % (95% CI):		
< 70	4.4 (3.6–5.1)	7.4 (6.1–8.1)
70–199	14.8 (13.5–16.1)	12.6 (11.4–13.8)
200–399	23.5 (21.8–25.0)	22.0 (20.2–23.2)
400–899	18.7 (17.1–20.1)	29.4 (27.6–30.9)
≥ 900	38.6 (36.8–40.5)	28.6 (26.3–30.3)
Time in ambulance (minutes), median (IQR):	46 (34–61)	47 (36–61)

*Abbreviations*: *CI* confidence interval, *CCI* Charlson Comorbidity Index, *ICD-10* 10th version of the International Classification of Diseases, *Cum.* cumulative, *IQR* interquartile range

**Table 2:** Pain scores and vital parameters of included patients

Treatment protocol	Standard (2 μg/kg)	Liberal (3 μg/kg)
*Pain characteristics for patients with complete pain scores:*	*n* = 1884	*n* = 2082
Pain, NRS score (mean, 95% CI):		
Before treatment initiation	7.59 (7.50–7.67)	7.45 (7.36–7.54)
At hospital arrival	4.36 (4.26–4.46)	4.08 (3.98–4.17)
NRS ≤ 3 at hospital arrival, n:		
No	1180	1167
Yes	704	915
NRS ≤ 3 at hospital arrival, % (95% CI)	37.4 (35.2–39.6)	44.0 (41.8–46.1)
Difference, ΔNRS (95% CI):		
Absolute decrease, units	3.23 (3.12–3.34)	3.37 (3.27–3.48)
Relative decrease, %	40.5 (39.0–42.1)	43.0 (41.3–44.7)
Cum. dose (μg, mean 95%CI) fentanyl and:		
NRS ≤ 3 at hospital arrival	102.9 (98.4–107.3)	105.9 (102.4–109.4)
NRS ≥ 4 at hospital arrival	126.9 (123.0–130.7)	136.3 (131.9–140.8)
*Characteristics of vital parameters for all included patients:*	*n* = 2598	*n* = 2680
GCS < 15, n (%):	68 (2.7)	69 (2.6)
Respiratory rate < 10/min, n (%):	9 (0.3)	10 (0.4)
Oxygen saturation < 90%, n (%):	547 (21.1)	608 (22.7)
MAP < 70 mmHg and decrease ≥ 10 mmHg, n (%):	182 (7.0)	199 (7.4)

*Abbreviations*: *CI* confidence interval, *IQR* interquartile range, *Cum.* cumulative, *NRS* numeric rating scale (0–10), Δ: difference

**Table 3:** Odds ratios for differences in effect and safety measures

	Odds ratio (95% CI)		*P*-value
	Crude	Adjusted^a^	
NRS ≤ 3 at hospital arrival:			
Standard	1 (reference)	1 (reference)	0.001
Liberal	1.42 (1.13–1.80)	1.47 (1.17–1.84)	
NRS decrease (relative) during transport:			
Standard	1 (reference)	1 (reference)	0.14
Liberal	1.15 (0.90–1.46)	1.18 (0.95–1.48)	
GCS < 15:			
Standard	1 (reference)	1 (reference)	0.76
Liberal	0.87 (0.27–2.80)	0.84 (0.27–2.62)	
Respiratory rate < 10/min:			
Standard	1 (reference)	1 (reference)	0.85
Liberal	1.07 (0.37–3.09)	1.11 (0.40–3.09)	
Oxygen saturation < 90%:			
Standard	1 (reference)	1 (reference)	0.15
Liberal	1.19 (0.96–1.47)	1.17 (0.95–1.44)	
MAP < 70 mmHg and decrease ≥ 10 mmHg:			
Standard	1 (reference)	1 (reference)	0.96
Liberal	1.08 (0.74–1.57)	1.01 (0.70–1.47)	

*Abbreviations*: *CI* confidence interval, *NRS* numeric rating scale, *GCS* Glasgow Coma Scale, *MAP* mean arterial pressure, *EMT* emergency medical technician, *PM* paramedic

^a^: Adjusted for age, sex, comorbidity, etiology (injury yes/no), time in ambulance, station size, ambulance personnel experience (EMT or PM) and mean dose administered by ambulance personnel. For vital parameters, the highest cluster level (ambulance station) has been removed in the adjusted analyses to ensure better model fit

The mean cumulative dose of fentanyl administered by EMTs/PMs in the liberal protocol was slightly higher than that of the standard protocol [117.7 μg (95% CI 116.7–118.6) vs. 111.5 μg (95% CI 110.7–112.4), *P* = 0.0001]. In the one-year period before the protocol change.

no difference existed between the two groups compared [106.7 μg (95% CI 105.7–107.7) vs. 108.0 μg (95% CI 107.0–109.0), *P* = 0.09]. Higher doses were given in the liberal treatment group for the patients not having sufficient pain relief (NRS > 3) at hospital arrival than in the standard treatment group [136.3 μg (95% CI 131.9–140.8) vs. 126.9 μg (95% CI 123.0–130.7) (*P* = 0.001)] (Table 2).

A considerable number of patients in both groups had missing pain scores [liberal: 22.7 (95% CI 21.1–24.3) vs. standard: 27.8 (95% CI 26.1–29.5), *P* = 0.001]. Characteristics of patients with missing pain scores are presented in Table 4. Generally, patients with missing pain scores were older, more comorbid, lower initial pain scores, and received lower doses of fentanyl compared with patients with complete information on pain scores. For the patients with missing pain scores, no differences in patient characteristics were observed between the standard treatment group and the liberal treatment group.

**Table 4:** Characteristics of patients with and without complete pain scores

Treatment protocol	Patients with missing pain scores		Patients with complete pain scores	
	Standard (2 μg/kg) (*n* = 714)	Liberal (3 μg/kg) (*n* = 598)	Standard (2 μg/kg) (*n* = 1884)	Liberal (3 μg/kg) (*n* = 2088)
Age, years (95% CI)	59.5 (57.7–61.2)	60.7 (58.7–62.7)	55.1 (54.1–56.1)	55.8 (54.8–56.7)
Male sex, % (95% CI)	43.7 (40.0–47.4)	45.8 (41.7–49.9)	46.0 (43.7–48.3)	48.8 (46.6–50.9)
CCI score, % (95% CI)				
0	55.2 (51.4–58.9)	52.0 (47.9–56.1)	61.4 (59.1–63.6)	58.4 (56.2–60.5)
1	17.6 (14.9–20.6)	16.7 (13.8–20.0)	13.9 (12.3–15.5)	14.6 (13.1–16.1)
2	9.4 (7.3–11.8)	11.4 (8.9–14.2)	10.2 (8.9–11.7)	10.7 (9.5–12.2)
3+	17.8 (15.0–20.8)	19.9 (16.8–23.3)	14.5 (13.0–16.2)	16.3 (14.8–18.0)
Time in ambulance (minutes), median (IQR):	44 (32–59)	48 (36–61)	47 (35–62)	47 (36–61)
Cum. dose (μg) fentanyl, mean (95% CI)	96.7 (92.3–101.0)	99.3 (94.6–104.0)	117.9 (114.9–120.9)	122.9 (119.9–125.9)
Initial NRS score^a^ (mean, 95% CI):	6.52 (6.24–6.79)	6.21 (5.91–6.51)	7.59 (7.50–7.67)	7.45 (7.36–7.54)

*Abbreviations*: *CCI* Charlson Comorbidity Index, *Cum.* cumulative, *NRS* numeric rating scale

^a^Given for the patients with an initial pain score but not a subsequent pain score at hospital arrival, which accounts for 307 patients in the standard group and 292 in the liberal group

## Discussion

In this large two-armed, cluster-randomised controlled trial we found that patients treated with a liberal protocol were more likely to have sufficient pain relief at hospital arrival than patients treated with a standard treatment protocol. It can be argued that the difference was small, and the question remains why more than half the patients had insufficient pain relief, even with a liberalised protocol. The explanation may be found in the conservative doses of administered fentanyl, which again may reflect concerns of malingering patients, apprehension of inducing side effects, and worries about blurring symptoms and further diagnostics [42, 43]. The same concerns may have arisen in other prehospital studies in which higher doses (120–220 μg) were only obtained when administered by physicians or Australian/American paramedics without dosing restrictions (Table 5). No differences in the occurrence of abnormal vital parameters were observed between the two groups and none of the patients were given naloxone, which suggests that EMTs/PMs can safely administer fentanyl in a liberalised protocol.

**Table 5:** Cumulative doses of opioids administered in prehospital studies

Author, year, country	Setting and providers	Design and sample	Adm. form	Opioid(s)	Protocol restrictions	Cumulative Doses	Equianalgesic i.v. Morphine dose
Krauss et al. [17] USA 2011	HEMS BLS and ALS (paramedic)	Single arm *n* = 500	i.v.	Fentanyl	5 μg/kg/hour	3.0 μg/kg	22.5 mg^a^
Bounes et al. [9] 2010 France	Two-tiered BLS and ALS (physician)	RCT *n* = 108	i.v. i.v.	Morphine Sufentanil	None	0.3 mg/kg 0.225 μg/kg	22.5 mg^a^
Bounes et al. [10] 2007 France	Two-tiered BLS and ALS (physician)	RCT *n* = 106	i.v. i.v.	Morphine Morphine	None	0.225 μg/kg 0.3 mg/kg	22.5 mg^a^
Bounes et al. [39] 2011 France	Two-tiered BLS and ALS (physician)	Observational *n* = 277	i.v. i.v.	Morphine Sufentanil	N/A	0.3 mg/kg^b^ 0.23 μg/kg^b^	22.5 mg^a^ 17.3 mg^a^
Galinski et al. [7] 2005 France	Two-tiered BLS and ALS (physician)	RCT *n* = 54	i.v. i.v.	Morphine Fentanyl	None	16 mg 150 μg	16 mg 15 mg
Albrecht et al. [5] 2013 Switzerland	HEMS BLS and ALS (physician)	Single arm *n* = 1202	i.v.	Fentanyl	None	157 μg	15.7 mg
Auffret et al. [46] 2014 France	Two-tiered BLS and ALS (physician)	RCT *n* = 85	i.v. i.v.	Morphine Midazolam +Morphine	None	15.5 mg 14.1 mg	15.5 mg 14.1 mg
Galinski et al. [11] 2007 France	Two-tiered BLS and ALS (physician)	RCT *n* = 65	i.v. i.v.	Morphine Ketamine+ Morphine	None	0.202 mg/kg 0.149 mg/kg	15.2 mg^a^
Jennings et al. [8] 2007 Australia	Two-tiered BLS and ALS (paramedic)	RCT *n* = 135	i.v. i.v.	Morphine Ketamine +Morphine	None	15 mg 35 mg	15 mg
DeVellis et al. [64] 1998 USA	HEMS BLS and ALS (paramedic)	Single arm *n* = 130	i.v.	Fentanyl	None	144 μg	14.4 mg
Oberholzer et al. [29] 2017 Switzerland	HEMS BLS and ALS (physician)	Single arm *n* = 778	i.v. i.v. i.v.	Fentanyl Morphine Ketamine	None	140 μg 7 mg 58 mg	14 mg 7 mg
Johansson et al. [65] 2009 Sweden	Two-tiered BLS and ALS (nurse)	Non-RCT *n* = 27	i.v. i.v. i.v.	Morphine Ketamine +Morphine	0.2 mg/kg	13.5 mg 27.9 mg 7.0 mg	13.5 mg
Frakes et al. [66] 2009 USA	HEMS BLS and ALS (paramedic)	Single arm *n* = 209	i.v.	Fentanyl	5 μg/kg/hour	1.7 μg/kg	12.8 mg^a^
McRae et al. [48] 2015 Australia	Two-tiered BLS and ALS (paramedic)	RCT *n* = 24	i.v. reg.	Morphine Regional Block+ Morphine	0.5 mg/kg	7.5 mg + 5 mg^b^ 5 mg^b^	12.5 mg 5 mg
Frakes et al. [67] 2006 USA	HEMS BLS and ALS (paramedic)	Single arm *n* = 100	i.v.	Fentanyl	5 μg/kg	1.6 μg/kg	12 mg^a^
Kanowitz et al. [16] USA 2006	Two-tiered BLS and ALS (paramedic)	Single arm *n* = 2129	i.v.	Fentanyl	None	118 μg	11.8 mg
Friesgaard et al. Denmark 2017	Two-tiered BLS and ALS (EMT-I)	cRCT *n* = 5278	i.v. i.v.	Fentanyl Fentanyl	3 μg/kg 2 μg/kg	117.7 μg 111.5 μg	11.8 mg
Silfvast et al. [13] 2001 Finland	Two-tiered BLS and ALS (physician)	RCT *n* = 36	i.v. i.v.	Morphine Alfentanil	10 mg 1 mg	10 mg 1 mg	10 mg 9–15 mg
Eidenbenz et al. [28] 2016 Switzerland	HEMS BLS and ALS (physician)	Single arm *n* = 1156	i.v. i.v. i.n.	Fentanyl Ketamine Fentanyl	None	100 μg^b^ 30 mg 100 μg	10 mg
Smith et al. [6] 2010 USA	HEMS BLS and ALS (physician)	RCT *n* = 200	i.v. i.v.	Morphine Fentanyl	20 mg 250 μg	4 mg 100 μg	4 mg 10 mg
Soriya et al. [68] 2011 USA	Two-tiered BLS and ALS (paramedic)	Before after *n* = 763	i.v.	Fentanyl	100 μg	100 μg	10 mg
Richard Hibon et al. [69] 2008 France	Two-tiered BLS and ALS (physician)	Before after *n* = 216	i.v.	Morphine	None	9.5 mg	9.5 mg
Fleischman et al. [35] 2010 USA	Two-tiered BLS and ALS (paramedic)	Before after *n* = 718	i.v. i.v.	Morphine Fentanyl	20 mg 200 μg	7.7 mg 92 μg	7.7 mg 9.2 mg
Friesgaard et al. [19] 2016 Denmark	Two-tiered BLS and ALS (EMT-I)	Single arm *n* = 2348	i.v.	Fentanyl	2 μg/kg	90 μg^b^	9.0 mg^b^
Galinski et al. [36] 2010 France	Two-tiered BLS and ALS (physician)	Observational *n* = 2279	i.v.	Morphine	N/A	9.0 mg	9.0 mg
Lebon et al. [70] 2016 Canada	Two-tiered BLS and ALS (paramedic)	Single arm *n* = 288	s.c.	Fentanyl	3 μg/kg	1.2 μg/kg	9 mg
Jennings et al. [58] 2010 Australia	Two-tiered BLS and ALS (paramedic)	Observational *n* = 315,273	i.v. i.v. inh.	Fentanyl Morphine Methoxyflurane	N/A	83.5 μg 7.6 mg 3.2 ml	8.4 mg 7.6 mg
Bakkelund et al. [71] 2013 Norway	Two-tiered BLS and ALS (EMT)	Single arm	i.v.	Morphine	N/A	7.5 mg^b^	7.5 mg^b^
Ricard-Hibon et al. [51] 1999 France	Two-tiered BLS and ALS (physician)	Before after *n* = 213	- i.v.	Analgesics Morphine	None	7.2 mg	7.2 mg
Brown et al. [53] 2016 USA	Two-tiered BLS and ALS (paramedic)	Before after *n* = 2128	i.v. i.v.	Morphine Morphine	20 mg	6.0 mg 7.1 mg	6.0 mg 7.1 mg
Fullerton-Gleason et al. [55]	Two-tiered BLS and ALS (paramedic)	Before after	i.v. i.v.	Morphine Morphine	N/A	6.6 mg 6.7 mg	6.6 mg 6.7 mg
Zedigh et al. [47] 2010 Sweden	Two-tiered BLS and ALS (nurse)	RCT *n* = 164	i.v. i.v.	Morphine Metoprolol +Morphine	5 mg 5 mg	5 mg 5 mg	5 mg 5 mg
Weldon et al. [12] 2016 Canada	Two-tiered BLS and ALS (paramedic)	RCT *n* = 207	i.v. i.v.	Morphine Fentanyl	20 mg 100 μg	4,25 mg 42,5 μg	4,25 mg 4,25 mg
Bruns et al. [72] 1992 USA	Two-tiered BLS and ALS (paramedic)	Observational *n* = 89	i.v.	Morphine		4 mg	4 mg
Middleton et al. [61] 2010 Australia	Two-tiered BLS and ALS (paramedic)	Observational *n* = 42,844	i.v. i.n. inh.	Morphine Fentanyl Methoxyflurane	0.5 mg/kg None	N/A	
Bendall et al. [73] 2011 Australia	Two-tiered BLS and ALS (paramedic)	Single arm *n* = 97,705		Morphine Fentanyl Methoxyflurane	N/A	N/A	
Thomas et al. [18] USA 2005	HEMS BLS and ALS (paramedic)	Single arm *n* = 177	i.v.	Fentanyl	5 μg/kg/hour	N/A	
Tran et al. [45] 2014 Vietnam	Rural, low resource setting	cRCT *n* = 308	i.m. i.v.	Morphine Ketamine	10 mg 0.2–0.3 mg/kg	N/A	
Rickard et al. [15] 2007 Australia	Two-tiered BLS and ALS (paramedic)	RCT *n* = 258	i.v. i.n.	Morphine Fentanyl	15 mg 300 μg	N/A	
Vergnion et al. [14] Belgium	N/A	RCT *n* = 101	i.v. i.v.	Morphine Tramadol	20 mg 200 mg	N/A	

*Abbreviations*: *N/A* Not applicable, *i.v.* intravenous, *i.m.* intramuscular, *RCT* randomised controlled trial, *cRCT* cluster randomised controlled trial, *i.n.* intranasal, *inh.* inhalation, *s.c.* subcutaneous, *BLS* basic life support, *ALS* advanced life support, *HEMS* helicopter-based emergency medical services, *Adm.* administration, *reg.* regional, *EMT-I* emergency medical technician - intermediate

^a^Assuming an average patient weight equal to 75 kg

^b^Median dose given

Few other randomized controlled trials on prehospital analgesia for adults have been conducted including small samples ranging from 24 to 312 patients. Similar to our study, two randomised controlled trials have investigated the impact of increasing the opioid doses on analgesic efficacy and safety. Woollard et al. investigated 172 prehospital patients with diverse aetiologies of pain. The researchers found that a rapid administration regiment leading to higher cumulative doses (14.8 mg) of intravenous nalbuphine (a semi-synthetic opioid) was more effective than a cautious regiment (10.7 mg) with a lower dose (ΔNRS: 4.29 units vs. 3.49 units, *P* = 0.028). In terms of side effects, a higher frequency of drowsiness in the patients treated with the rapid regiment was observed [44]. Bounes et al. reached the opposing conclusion in a study on 106 prehospital patients with acute pain, finding no superior analgesic effect or difference in safety parameters for a higher fixed dose of intravenous morphine compared to a lower fixed dose (absolute doses not given) [10].

Other studies have compared newer synthetic opioids with intravenous morphine. Smith et al. investigated 204 trauma patients in a physician-staffed helicopter and found no difference in analgesic effect, occurrence of abnormal vital parameters or adverse effects when compared with intravenous fentanyl [6]. A smaller physician-based study on a diverse sample of 60 patients in France demonstrated no difference between intravenous fentanyl and morphine in terms of efficacy, vital sign abnormalities and mild adverse effects [7]. Attempting to find a faster onset of action for intravenous fentanyl in 207 patients with ischemic type chest pain, Weldon et al. found no analgesic superiority compared with intravenous morphine. Also, no differences in vital signs or adverse effects was found [12]. Another small study of 36 patients with ischemic type chest pain found more rapid onset and more effective pain relief of intravenous alfentanil than intravenous morphine and no differences in vital signs [13]. Investigating another fast-acting opioid, Bounes and colleagues found no superior analgesic effect of intravenous sufentanil compared to intravenous morphine for 108 trauma patients; in addition, vital signs and adverse effects were similar [9]. Other randomised controlled trials have assessed the impact of ketamine [45], combinations of morphine and ketamine [8, 11] or other combinations [46, 47] or regional nerve blockades [48, 49]. Most of the trials have been inconclusive, underpowered and with varying levels of quality, which probably reflect the jurisdictional and practical challenges of conducting research in an austere prehospital environment [50]. For the studies demonstrating analgesic superiority [8, 11, 13, 44], the frequency of adverse effects was also higher, so neither specific conclusions nor recommendations can be made. Few other non-randomised (before-after) trials have sought to optimise prehospital pain management by modifying existing practice and educating the involved healthcare providers. These studies all found insignificant changes in pain scores, pain score documentation, and/or the cumulative opioid doses provided [35, 51–57].

### Strengths and limitations

The strengths of this two-armed, cluster-randomised controlled trial rest on the large sample size and the real-world population-based prehospital data individually merged with validated national registries [24, 25]. We mitigated the risk of unobserved secular effects on estimates by adding a non-historical control group and adopting a pragmatic, cluster-randomised design, reflecting a real-life delivery of intervention. We undertook a stratified randomisation in order to mitigate the risk of unbalanced patient samples and cluster-specific characteristics at the ambulance station level. However, the study also has a number of limitations that need to be addressed.

First of all, similar to other prehospital investigations into pain management, pain scores were missing in our study (up to 27.8%) and this may have introduced bias. Depending on study design and patient selection, the proportion of patients with missing pain data in other prehospital studies ranges from 15% [58], 25–35% [5, 28, 35, 36, 38], 40–60% [30, 59–61] to 70–80% [52, 62]. Missing data on pain scores impose a potential threat to the validity of the findings and should be taken into account when interpreted. We presented characteristics of patients with and without pain scores and found that patients with incomplete pain scores were older, had more comorbidities and received lower doses of fentanyl.

Second, the implementation of the liberal protocol was left to the discretion of the enrolled ambulance station leaders. This approach thus reflects the effect of protocol changes when implemented in real-life settings but may also partly explain the relatively small difference observed between the groups.

Third, our study was probably not powered to detect small differences on abnormal vital parameters as proxies of adverse effects, as these events are infrequent, and therefore a risk of type II errors exists. As the most conservative example, 7093 patients in each arm would have been required in order to find an occurrence of hypotension of 3% among patients treated under the standard protocol compared to 4% among patients treated under the liberal treatment protocol.

Last, the occurrence of fentanyl side effects can be difficult to quantify precisely with discrete measures. Exemplified by respiratory depression, hypoxemia may very well be correlated with oxygenation as measured by pulse oximetry when patients are breathing at atmosphere oxygen levels. However, it will be affected by factors such as supplemental oxygen therapy or peripheral vasoconstriction [63]. The presence or absence of abnormal vital parameters should therefore be interpreted in the light of these precautions.

## Conclusion

Liberalising an intravenous fentanyl treatment protocol applied by EMTs/PMs resulted in slightly more patients having sufficient pain relief at hospital arrival compared to patients treated under a standard treatment protocol. Fentanyl doses were conservatively administered in both groups and the high overall proportion of patients with insufficient pain relief suggests that more should be done to ensure protocol adherence. No differences in the occurrence of abnormal vital parameters were observed between the two groups, suggesting that future efforts in optimising intravenous fentanyl protocols for non-physician staff can be made safely under ongoing evaluation and monitoring of the patients.

## Abbreviations

95% CI: 95% confidence interval
CCI: Charlson comorbidity index
EMCC: Emergency Medical Coordination Centre
EMT: Emergency medical technician
GCS: Glasgow Coma Scale
ICC: Intracluster correlation
ICD: International Classification of Diseases
MAP: Mean arterial pressure
NRS: Numeric rating scale
OR: Odds ratio
PM: Paramedic
